# Comparative efficacy of a novel *Bacillus subtilis*-based probiotic and pharmacological zinc oxide on growth performance and gut responses in nursery pigs

**DOI:** 10.1038/s41598-023-31913-0

**Published:** 2023-03-22

**Authors:** Sudhanshu Sudan, Lauren Fletcher, Xiaoshu Zhan, Serena Dingle, Rob Patterson, Lee-Anne Huber, Robert Friendship, Elijah G. Kiarie, Julang Li

**Affiliations:** 1 Department of Animal Biosciences, University of Guelph, Guelph, ON Canada; 2 Department of Population Medicine, University of Guelph, Guelph, ON Canada; 3 CBS Bio Platforms Inc, Calgary, AB Canada

**Keywords:** Biotechnology, Microbiology

## Abstract

In this study, we assessed the efficacy of a novel *Bacillus subtilis* probiotic in improving growth performance and gut responses in comparison to pharmacological zinc oxide (ZnO) in nursery pigs. A total of 96 piglets were randomly assigned to four groups: Negative control (NC), Positive control (PC, 3000 mg Zn /kg feed), *B.subtilis* low dose (BS9-L, 2 × 10^7^ CFU/pig) and *B.subtilis* high dose (BS9-H, 2 × 10^9^ CFU/pig). Growth performance, diarrhea rate, gut mucosal gene expression and fecal microbial populations were evaluated. *B.subtilis* administration did not improve piglet bodyweight. BS9-L showed (P < 0.05) higher average daily gain (ADG) in Period 2 (D14-D28). BS9 groups had (P < 0.001) lower feed conversion ratio (FCR) in Period 2 (D14-D28) and overall. Like the ZnO-group, BS9 groups had lower (P < 0.01) diarrhea rate. A significant reduction (P < 0.05) in fecal *E. coli*, total coliforms, and an increase in lactic acid bacteria and *Bacillus spp.* in BS9 groups was observed. BS9 group had reduced (P < 0.05) mRNA levels of intestinal IL-8 and higher levels of *MUC-1* and occludin and *TJP-1* compared to negative control. These findings suggest that probiotic BS9, may promote growth performance, and ameliorate various indicators of intestinal health in piglets. Hence, it may serve as a prospective alternative to ZnO growth promoter in commercial swine production.

## Introduction

Post-weaning diarrhea (PWD) caused by enterotoxigenic *Escherichia coli* is a common and economically important health problem in swine production. This disease is characterised by loss of body weight and increased mortality^1^. Traditionally, antibiotics have been used as an effective mitigation strategy to combat PWD, however, due to rise in antibiotic resistant bacteria associated with livestock farming, their use is heavily discouraged, including some countries bannig their use as growth promoters in animal feedstuff^2^. Additionally, pharmacological levels of heavy metals particularly zinc oxide (ZnO) has proven to be effective in control of PWD when included at levels > 2500 ppm have also come under considerable scrutiny due to their negative impact on the animal health seen via accumulation of heavy metal in the organs (such as pancreas and liver) and the environment via soil and water^3–5^. Additionally, higher doses of ZnO have been shown to negatively impact intestinal microbiota in piglets, for example decreasing abundance of lactic acid producing bacteria, and increasing *Enterobacteriaceae* and other less prominent species, directly impacting the metabolic response^6–8^. Thus, developing an alternative and environmentally friendly strategy to combat PWD and improve overall health of pigs is imperative.

Growing evidence suggests that inclusion of probiotic bacteria can greatly benefit piglets during weaning by improving their nutrient digestibility^9^, reducing pathogen load^1^, strengthening mucosal physiology^10^, and improving gut immunity^11,12^, microbiota diversity and overall growth performance^9,12^. Additionally, since gut microbiota play a significant role as a first line of defence against ingested pathogens, studies suggest that inclusion of specific probiotics in the diet may help reduce intestinal colonisation by antibiotic-resistant bacteria^13^. Notably, lactic acid bacteria such as lactobacillus have been extensively studied for their probiotic potential and have successfully made their way to commercial applications. Comparatively, studies on *Bacillus* species, such as *Bacillus subtilis* have recently gained more attention. The ability to form spores at high temperatures and survive low pH environments make *Bacillus subtilis* a robust strain to be developed as an in-feed probiotic supplement^14^. In light of PWD, recent studies have indeed shown that *Bacillus subtilis*-based probiotic supplementation (commercially available and newly isolated) reduced the incidence and severity of diarrhea and enhanced growth performance by modulating intestinal microbiota and enhancing systemic immunity in weanling piglets experimentally infected with enterotoxic *Escherichia coli* (ETEC)^15,16^. Additionally, weaning associated diarrhea can also be triggered via use of economical diets which are mostly plant based (Corn- and soybean meal-based) are less costly but have been associated with lower intestinal integrity and greater diarrhea incidence^17–19^. Since, many *Bacillus-*based probiotics exhibit exogenous digestive enzyme activity, inclusion of such probiotic strains to nursery pigs may help alleviate the feed-induced diarrhea incidence and maintain or improve growth performance, which may provide an economical feeding strategy that could benefit producers globally. We recently isolated a novel *Bacillus subtilis* strain (BS9) which showed enhanced probiotic properties including strong protease, cellulase and cytoprotective activities, compared to the commercially available *Bacillus-*based probiotic^20,21^. In this study, we evaluated and compared the efficacy of a novel *Bacillus*-based probiotic, BS9 as alternative to pharmacological zinc in piglets fed a simple corn- and soybean-meal based diet. Additionally, since, targeted probiotics necessitate additional knowledge of host and probiotics interactions, we also analysed the differences in intestinal metabolic signatures upon BS9 supplementation and metabolic pathways influenced by the probiotic inclusion.

## Materials and methods

All experiment procedures were reviewed and approved by the University of Guelph Animal Care Committee, and the pigs were cared for as per the guidelines set forth by Canadian Council on Animal Care^22^. The experimental protocols and methods in this study were carried out in compliance with the ARRIVE guidelines.

### Experimental design and dietary treatments

A total of 96 crossbred piglets (Landrace × Yorkshire × Duroc, 6.4 ± 0.5 kg BW) with equal number of gilts and barrows selected from the Arkell Swine Research Facility, University of Guelph, Ontario, Canada were randomly assigned into four groups immediately after weaning: Negative control group (a basal diet; NC), Positive control group (a basal diet with 3000 mg zinc/kg, PC), BS9-L group (a basal diet; provided with low dose, 2 × 10^7^ CFU B. *subtilis*-9/pig) and BS9-H group (a basal diet; provided with high dose, 2 × 10^9^ CFU *B. subtilis*-9/pig). All groups had six replicate pens with four piglets per replicate (2 gilts and 2 barrows). The corn- and soybean meal-based basal diet was formulated to meet the estimated nutrient requirements established by the National Research Council for nursery pigs (Supplement Table S1)^23^. The diets were fed in two phases across the periods: phase I (week 1) and phase II (weeks 2–4). The novel *B. subtilis-*9 doses were administered through water twice per day (morning and evening) for 21 days. Each pen was equipped with a drinker and feeder, and piglets had ad libitum access to feed and water throughout the trial. The trial lasted for 28 days.

### Assessment of growth performance and health status

All piglets were weighed individually at days 0, 7, 14, 21, and 28 during the trial. The feed consumption per pen was monitored weekly. Average daily gain (ADG), average daily feed intake (ADFI), and feed conversion ratio (FCR) were calculated for the periods of days 1–14, 15–28 and 1–28 of the trial. The health status of piglets during the trial was assessed by fecal consistency scoring using a four-grade system, where 0 corresponded to firm and dry, 1 to pasty, 2 to thick and fluid, and 3 to watery^24^. Pen was considered the experimental unit (n = 6). Weekly fecal scores were obtained by adding daily fecal consistency scores from all pens. The occurrence of diarrhea was defined as maintaining a score of 3 for at least 1 day. The incidence of diarrhea (%) was calculated by dividing the total number of diarrheal piglets over a period by the number of piglets and days in that period multiplied by 100.

### Sample collection and preparation

At days 3, 7, 14 and 28 fresh fecal samples were collected (one pig per pen, n = 6) by using sterile anal swabs in phosphate buffered saline (PBS). Samples were transported to the lab and analysed the same day for microbial populations. At day 28, one pig per pen (n = 6) was euthanized with an IV injection of 3 mL of Pentobarbitol (250 mg/mL; Euthasol, Virbac, TX. Samples of colon contents were collected, snap frozen in liquid nitrogen and stored in – 80 °C ultra-low temperature freezer until further use for metabolomic analyses. Approximately 2 cm of ileum tissue samples were collected, washed with sterile PBS, and stored in respective 2 ml centrifuge tubes containing RNAlater™ Stabilization Solution (Invitrogen, Mississauga, ON, Canada) at − 80 °C in an ultra-low temperature freezer for gene expression analysis. Blood samples were collected in 15 mL centrifuge tubes by orbital sinus bleeding technique by trained technician. Blood samples were centrifuged (3000×*g* for 10 min at 4 °C) on the same day to collect plasma, which was aliquoted and stored in – 80 °C ultra-low temperature freezer until further analysis.

### Determination of fecal microbial populations

Population analysis on the microbial communities was performed using species selective medium method as previously described^25–27^. Briefly, fresh fecal samples (anal swabs in PBS) were obtained from one pig per pen (n = 6), serially diluted (tenfold) and seeded on plates containing selective growth medium DeMan, Rogosa and Sharpe (MRS, Oxoid, Thermo Fisher Scientific, ON, Canada) for total lactobacillus, Violet Red Bile-based 3 M™ Petrifilm™ E. coli/Coliform petrifilm plates (3 M, London, ON, Canada) for *E. coli* and other coliforms and polymyxin pyruvate egg yolk mannitol bromothymol blue Agar (PEMBA) for *Bacillus spp*. The plates were incubated aerobically in 37º C incubator for 24–48 h, after which microbial populations were determined via counting colony forming units (CFU).

### Analysis of mRNA levels of genes related to intestinal health

Gene transcript levels were analysed using real-time polymerase chain reaction (RT-PCR), as previously described^28^. Briefly, total RNA was extracted from the ileum tissue using Norgen Total RNA purification kit (Norgen Biotek, Thorold, ON, Canada) and following manufacturer’s instructions. The concentration and 260/230 ratio of the total RNA was measured using a NanoDrop 8000 (Thermo Fisher, Cat#: ND8000-GL, Waltham, MA, USA) to ensure the samples were optimal for reverse transcription. Total RNA samples were reverse transcribed into complementary DNA using iScript reverse transcription supermix kit (Bio-Rad, Mississauga, ON, Canada) following manufacturer’s instructions in the Thermal cycler (Bio-Rad, Mississauga, ON, Canada) at the following conditions: 25 °C for 5 min, 46 °C for 20 min, 95 °C for 1 min, and then held at 4 °C. Quantitative real time PCR (qPCR) was performed using Ssoadvanced™ Universal SYBR Green supermix (Bio-Rad, Mississauga, ON, Canada). Thermal cycling parameters were kept as 2 min at 95 °C for enzyme activation, 5 s at 95 °C for denaturation, and 20 s at 60 °C for annealing. RT-PCR was performed for 40 cycles in a Real-Time PCR System (CFX 96, Bio-Rad, Mississauga, ON, Canada). Primers were designed using Primer Blast tool (NCBI; National Center for Biotechnology Information) and synthesized by Integrated DNA Technologies (IDT, Coralville, IA, USA). Primer information is listed in the Supplement Table S2. After determining the primer efficiencies, relative mRNA abundance was analysed by normalizing the cycle threshold (CT) values of the target genes to the CT values of the housekeeping gene encoding GAPDH. The results are presented as fold change using the 2^−ΔΔCT^ method.

### Metabolomic analysis

To determine the effect of supplementation of *B*. s*ubtilis*-9 on the metabolomic profile in weaning piglets, we performed metabolomic analysis using ultra high-performance liquid chromatography (UHPLC) and mass spectrometer (MS) system (LC–MS). Briefly, digesta from the colon was extracted, snap frozen, and transferred in dry ice to the Biozone Mass Spectrometry Facility in the Chemical Engineering Department at the University of Toronto for metabolite extraction, and liquid chromatography-mass spectrometry (LC–MS) analysis (courtesy of metabolomics specialist, Robert Flick). Briefly, total protein was precipitated from the samples and metabolites were vacuum dried using a speedvac at ambient temperature, followed by resuspension in one tenth of the original volume using the appropriate starting solvent for each chromatography method. Analysis was performed using a Thermo Scientific Ultimate 3000 UHPLC (Thermo Fisher Scientific, Waltham, MA, USA) equipped with a Hypersil Gold C18 column (50 mm × 2.1 mm, 1.9 μm) (Thermo Scientific, Waltham, MA, USA) for Reverse Phase Chromatography and a Phenomenex Luna NH2 column (150 mm × 2 mm, 3 μm), both with guard columns for Normal Phase Chromatography. Column temperature was kept at 40 °C for both methods. Autosampler volume was kept at 5 °C and injection volume was set at 10 μL for both phases. Q-Exactive Orbitrap mass spectrometer (Thermo Fisher Scientific) equipped with a Heated Electrospray Ionization (HESI II) probe (pray Voltage: 3.5 kV; Capillary Temperature: 320 °C; m/z range: 80–1200; Resolution: 70,000) was used for compounds detection. The system was operated in negative and positive ionization modes for generating spectra. For the untargeted approach, data were processed (raw signals exacting, data baselines filtering, peak identification, and integration) and metabolite detection (KEGG and BioCyc database) using the differential analysis software package Compound Dis-coverer 2.1 (Thermo Scientific) with mass tolerance at 5 ppm. For targeted approach, peak area was used for identification by Xcalibur Qual Browser Software (Thermo Scientific) with mass tolerance at 5 ppm.

### Statistical analysis

Growth performance data were analysed by one way ANOVA post hoc Tukey test with pen as the experimental unit. For diarrhea score, individual pens (n = 6) were considered experimental units. Data were analyzed with GraphPad Prism v. 7.0 (GraphPad Software, Inc., San Diego, CA, USA) using one-way ANOVA with Tukey’s post hoc test and mixed general linear model curve fitting procedure. The P value < 0.05 was considered significant for all statistical tests and 0.05 ≤ P ≤ 0.10 was considered a significant trend.

Metabolomic data were analyzed by performing univariate and multivariate statistical analysis using Metaboanalyst (version 5.0) online analysis software (www.metaboanalyst.ca). Briefly, data were first filtered using interquantile range (IQR) to select only top maximum features. Processed data were log transformed and scaled before applying univariate and multivariate tests. Principal Component Analysis (PCA) was used to visualize the metabolic separation between the groups, and two sample t-tests and Wilcoxon rank sum tests were used to identify the significantly different metabolites contributing to this metabolic separation. P < 0.05 was considered significant for all statistical tests. Finally, enrichment analysis was performed in Metabolanalyst to identify the modulation in biological pathways upon probiotic supplementation.

## Results

### Impact of *B. subtilis*-9 supplementation on growth performance

No significant difference in the body weights was observed among the groups in the overall period (Table 1). However, in week 4, a significant increasing trend (P ≤ 0.1) was observed in the body weights of the piglets fed low and high doses of BS9.

**Table 1:** Weekly body weight of weaned pigs fed diets supplemented with BS9 (in kg).

Day	NC	PC	CP9-L	CP9-H	*P* value		
					*P*_*O*_	*P*_*L*_	*P*_*Q*_
Body weight (kg)							
D0	6.49 ± 0.17	6.43 ± 0.11	6.32 ± 0.06	6.23 ± 0.09	0.39	0.26	0.61
D7	6.6 ± 0.18	6.55 ± 0.11	6.5 ± 0.06	6.39 ± 0.09	0.59	0.52	0.50
D14	8.55 ± 0.19	8.82 ± 0.19	8.74 ± 0.13	8.34 ± 0.18	0.94	0.62	0.15
D21	11.22 ± 0.29	12.57 ± 0.59	11.96 ± 0.13	11.88 ± 0.33	0.14	0.03	0.66
D28	15.82 ± 0.31	16.75 ± 0.52	16.98 ± 0.19	16.85 ± 0.4	0.10	0.14	0.23
Average daily gain (kg/day)							
D 0–14	0.15 ± 0.004	0.18 ± 0.01	0.16 ± 0.01	0.15 ± 0.01	0.30	0.17	0.39
D 14–28	0.52 ± 0.01^a^	0.57 ± 0.02^ab^	0.59 ± 0.01^b^	0.58 ± 0.02^ab^	**0.03**	0.002	0.11
D 0–28	0.34 ± 0.01	0.37 ± 0.01	0.38 ± 0.02	0.37 ± 0.02	0.15	0.17	0.18
Average daily feed intake (kg/day)							
D 0–14	0.23 ± 0.01	0.25 ± 0.01	0.24 ± 0.02	0.23 ± 0.01	0.50	0.53	0.30
D 14–28	0.79 ± 0.04	0.87 ± 0.02	0.81 ± 0.02	0.78 ± 0.02	0.10	0.82	0.18
D 0–28	0.51 ± 0.02	0.56 ± 0.02	0.52 ± 0.02	0.51 ± 0.01	0.10	0.82	0.18
Feed conversion ratio							
D 0–14	1.60 ± 0.05	1.43 ± 0.08	1.41 ± 0.05	1.61 ± 0.15	0.36	0.45	0.27
D 14–28	1.51 ± 0.04^a^	1.52 ± 0.03^a^	1.38 ± 0.03^b^	1.3 ± 0.02^b^	** < 0.01**	0.73	< 0.01
D 0–28	1.53 ± 0.04^a^	1.51 ± 0.04^a^	1.38 ± 0.02^b^	1.34 ± 0.02^b^	** < 0.01**	0.59	< 0.01

Different superscript letters within a row represent statistically significant differences among groups (P < 0.05); P_O_ (Statistical difference), P_L_ and P_Q_ (Linear and quadratic effects of the three doses of probiotic BS9, respectively). Individual pens (n = 6/group) were considered a unit. Data are presented as mean ± standard error of the mean (SEM).

Significant values are given in bold.

There were no significant differences among the different groups for average daily feed intake (ADFI; Table 1). However, a significant increasing trend (P ≤ 0.1) was observed for PC group in week 4 and over all period (D0–D28). No improvement in the average daily gain (ADG) was observed among the groups in period 1 (D0–D14) and overall period (D0–D28). However, in period 2 (D14–D28), ADG of BS9-L group was significant higher (P < 0.05) compared to other groups (Table 1).

Feed conversion ratio (FCR) showed no significant difference among the different groups in period 1 (Table 1). However, in period 2 and overall, the FCR was significantly reduced (P < 0.0001) in the *bacillus*-fed groups, while no significant difference in FCR was observed among PC and NC groups in period 2 and overall. These results suggest that B*. subtilis*-9 supplementation may improve weaning piglet feed utilization efficiency and growth performance.

### Impact of *B. subtilis*-9 supplementation on fecal characteristics

Zinc Oxide and BS9-L group showed the lowest (P < 0.01) fecal score in week 1 (Table 2). During week 2 and 3, piglets in the NC group showed a significantly (P < 0.001 and < 0.01, respectively) higher fecal score compared to PC and *bacillus*-fed groups. No significant difference was observed in weekly fecal score among the groups during week 4, although a significant trend (P ≤ 0.1) was observed.

**Table 2:** Comparative analysis of fecal consistency of weaned pigs administered with BS9 or ZnO.

Period	NC	PC	BS9-L	BS9-H	*P* value		
					*P*_*O*_	*P*_*L*_	*P*_*Q*_
Average weekly fecal score							
Week 1	1.31 ± 0.14a	1.02 ± 0.7ab	0.90 ± 0.3b	0.74 ± 0.1b	< 0.01	0.03	< 0.01
Week 2	2.38 ± 0.07a	1.52 ± 0.17b	1.52 ± 1.3b	1.41 ± 1.19b	< 0.01	< 0.01	< 0.01
Week 3	1.19 ± 1.20a	0.62 ± 0.09b	0.67 ± 0.13b	0.60 ± 0.12b	< 0.01	< 0.01	0.12
Week 4	0.19 ± 0.06	0.07 ± 0.03	0.1 ± 0.03	0.05 ± 0.05	0.11	0.05	0.41
Diarrhea rate (%)							
D0–14	6.35 ± 0.66a	2.18 ± 0.59b	1.98 ± 0.61b	2.38 ± 0.72b	< 0.01	< 0.01	0.01
D14–28	0.60 ± 0.41	0.20 ± 0.20	0.20 ± 0.20	0.00	0.40	0.20	0.40
D0–28	3.47 ± 1.20a	1.19 ± 0.92b	1.09 ± 0.87b	1.19 ± 0.61b	< 0.01	< 0.01	0.01

Different superscript letters within a row represent statistically significant differences among groups (P < 0.05); P_O_ (Statistical difference), P_L_ and P_Q_ (Linear and quadratic effects of the three doses of probiotic BS9, respectively). Individual pens (n = 6/group) were considered a unit. Data are presented as mean ± standard error of the mean (SEM). Fecal consistency score of 0 correspond to firm and dry, 1 to pasty, 2 to thick and fluid and 3 to watery. Average weekly fecal scores were calculated by adding daily fecal consistency scores from all pens divided by 7. The diarrhea rate (%) was calculated by dividing the total number of diarrheal piglets over a period divided by the number of piglets and days in that period multiplied by 100.

Diarrhea rate significantly (P < 0.001) decreased in PC and *bacillus*-fed groups in Period 1 (D0-14) and overall (D0–28) compared to NC group (Table 2). No significant difference was observed between PC and *bacillus*-fed groups during these periods. Additionally, there was no significant difference in diarrhea rate in Period 2 (D14–D28). These data suggest that supplementation with novel B. *subtilis*-9 may improve the weaning- and diet- associated diarrhea comparable to ZnO supplementation.

### Impact of B. *subtilis*-9 supplementation on gut microbial characteristics

Piglets in the NC group showed a significant increase in the fecal *E. coli* and total coliform counts at all testing time points compared to the bacillus-fed groups (Table 3). Interestingly, piglets in NC group showed no significant difference in *E. coli* counts compared to piglets in the PC group at D3, D7 and D28. Piglets in both high and low dose bacillus-fed groups showed a significant decrease in the *E. coli* counts compared to the piglets in the PC group at all time periods tested, except for D14, where piglets in the BS9-H and PC groups had no significant difference in the *E. coli* counts (Table 3). Similarly, for total coliform counts, no significant difference was observed between piglets in the NC and PC groups, except for D7 and D14, where piglets in the PC group had lower total coliform counts than the NC group (Table 3). Piglets in both high and low dose bacillus-fed groups showed a significant decrease in the total coliform counts compared to the piglets in the PC group at all time periods tested, except for D14, where piglets in the BS9-L and PC groups had no significant difference in the total coliform counts (Table 3). These data suggest that supplementation with novel B. *subtilis*-9 may decrease the *E. coli* and total coliform counts in the weaning piglets, significantly better than ZnO supplementation.

**Table 3:** Fecal microbial counts (log^10^ CFU/ml) of weaned pigs fed diets supplemented with BS9.

Day	NC	PC	BS9-L	BS9-H	*P* Value
E. *coli*					
D3	6.54 ± 0.09^a^	4.54 ± 0.19^ab^	4.60 ± 1.17^ab^	2.81 ± 1.26^b^	0.03
D7	5.11 ± 0.12^a^	5.26 ± 0.93^a^	1.99 ± 1.26^b^	2.02 ± 1.28^b^	0.01
D14	5.46 ± 0.08^a^	2.40 ± 1.1^b^	1.42 ± 0.90^c^	2.12 ± 0.95^b^	0.01
D28	4.99 ± 0.13^a^	3.43 ± 1.0^a^	1.57 ± 0.99^b^	1.42 ± 0.91^b^	0.02
Total coliforms					
D3	6.33 ± 0.09^a^	6.11 ± 0.2^a^	3.85 ± 1.23^b^	2.81 ± 1.26^b^	0.03
D7	6.68 ± 0.11^a^	4.81 ± 0.98^b^	2.12 ± 1.34^c^	3.07 ± 1.38^c^	0.04
D14	5.62 ± 0.06^a^	2.96 ± 1.21^ab^	3.22 ± 1.02^ab^	1.50 ± 0.96^b^	0.02
D28	5.03 ± 0.13^a^	3.44 ± 1.09^a^	1.58 ± 0.99^b^	1.42 ± 0.91^b^	0.03
Lactic acid bacteria					
D3	5.58 ± 0.19	5.71 ± 0.17	5.65 ± 0.09	5.62 ± 0.15	0.95
D7	5.64 ± 0.09^a^	5.88 ± 0.20^ab^	6.24 ± 0.09^b^	6.27 ± 0.07^b^	0.01
D14	6.49 ± 0.09^ab^	6.19 ± 0.16^a^	6.81 ± 0.11^b^	6.78 ± 0.2^ab^	0.02
D28	6.31 ± 0.02^ab^	6.26 ± 0.08^a^	6.30 ± 0.06^ab^	6.6 ± 0.05^b^	0.01
Bacillus *spp.*					
D3	5.03 ± 0.05^ab^	4.95 ± 0.08^a^	5.20 ± 0.07^ab^	5.25 ± 0.04^b^	< 0.01
D7	5.11 ± 0.08^a^	5.26 ± 0.04^a^	5.45 ± 0.04^b^	5.51 ± 0.04^b^	< 0.01
D14	5.89 ± 0.09^a^	5.94 ± 0.17^ab^	6.04 ± 0.04^ab^	6.18 ± 0.11^b^	0.03
D28	6.27 ± 0.05^a^	6.23 ± 0.11^a^	6.39 ± 0.03^ab^	6.54 ± 0.07^b^	0.02

CFU, Colony forming units were populated using fecal swabs and microbial specific nutrient agar plates. Different superscript letters within a row represent statistically significant differences among groups (P < 0.05). Data (1 pig/pen, n = 6) are presented as mean ± standard error of the mean (SEM).

No significant difference in Day 3 fecal lactic acid bacteria (LAB) counts were noticed among the groups (Table 3). However, at D7, piglets in bacillus-fed high and low dose groups showed a significant increase (P < 0.005) in the LAB counts compared to piglets in the NC and PC group. Piglets in the PC group showed significantly lower LAB counts compared to piglets in the NC and bacillus-fed groups at D14 and D28. At D14 and D28, a significant increase in LAB counts were observed in the bacillus-fed piglets compared to piglets in the NC group, except for D28, where piglets in the BS9-L and NC groups had no significant difference in the LAB counts (Table 3). These data suggest that supplementation with B. *subtilis*-9 may increase the LAB counts in the weaning piglets, significantly greater than ZnO supplementation.

Overall, piglets in the BS9-H group showed significant increase in the *Bacillus spp.* counts compared to all the groups at all time points tested (Table 3). At D3 and D7, piglets in the BS9-L group showed significantly increased *Bacillus spp.* counts compared to the piglets in the NC and PC groups. However, by D14 and D28 no significant difference among piglets in the BS9-L and NC group was observed for *Bacillus spp.* counts (Table 3). These data suggest that supplementation with novel B. *subtilis*-9 may increase the *Bacillus spp.* counts in the weaning piglets, significantly better than ZnO supplementation.

### Impact of B. *subtilis*-9 supplementation on gut mucosa gene expression

**Figure 1:**
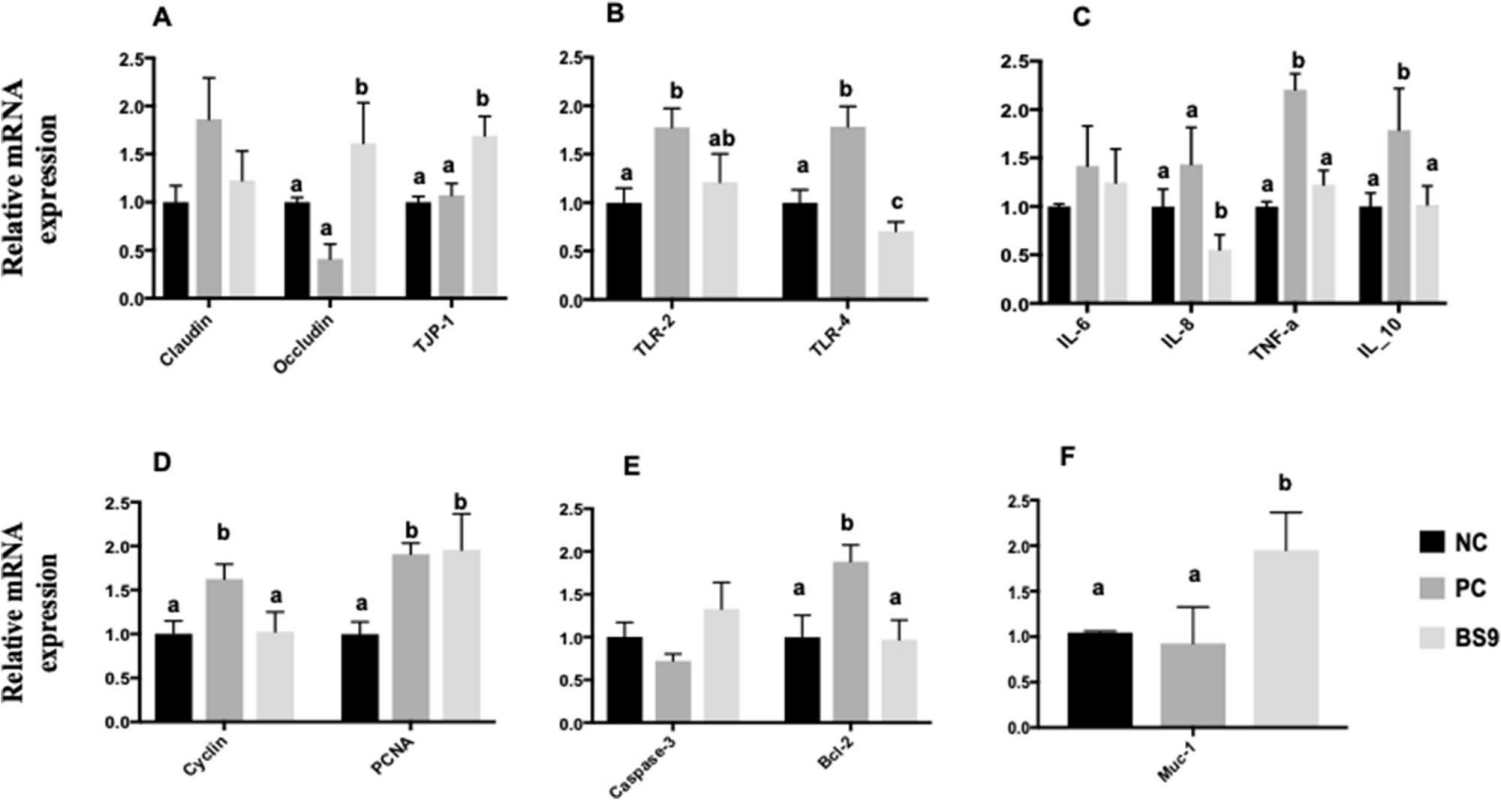
BS9 supplementation modulates gut mucosal gene expression in weaning piglets. At day 28, tissue samples were collected from BS9 supplemented and control diet groups and subjected to qPCR analysis. mRNA levels of gut health associated genes were detected. (**A**) modulation of tight junction genes, (**B**) modulation of Toll like receptors genes, (**C**) modulation of inflammation markers genes, (**D**) modulation of cell proliferation genes, (**E**) modulation of apoptosis related genes and (**F**) modulation of enteric mucin secretions genes. Expression level of all target genes was calculated relative to the housekeeping gene GAPDH using 2 − ΔΔCT method. Data are presented as means ± standard error of the mean (SEM) with n = 5 with atleast two technical replicates. Means marked with different letters (a, b, c) differ significantly at P values of < 0.05.

We first analysed impact of B. *subtilis*-9 supplementation on the transcript levels of tight junction genes in weaning piglets at the end of the trial (D28). Based on the results from the growth performance data, we selected BS9-L for the gene expression study. Piglets in the BS9 fed group showed significantly higher mRNA abundance of tight junction genes *occludin* and *TJP-1* compared to the piglets in the NC and PC group (Fig. 1A). No significant difference in the mRNA levels of tight junction genes was observed between piglets in the NC and PC group.

The impact of B. *subtilis*-9 supplementation on the intestinal expression of toll-like receptor genes and related inflammatory genes was also examined. Piglets in the BS9 fed group showed significantly lower mRNA levels of TLR-4 gene, but not TLR-2 gene as compared to piglets in the NC and PC group (Fig. 1B). Piglets in the PC group showed a significantly higher mRNA levels of TLR-2 and TLR-4 genes compared to piglets in the BS9 fed and NC group.

No significant difference in the mRNA levels of proinflammatory genes IL-6 and TNF-α and anti-inflammatory gene IL-10 was observed among piglets in the NC and BS9 fed group (Fig. 1C). However, a significant decrease in the mRNA levels of proinflammatory genes IL-8 was observed in the BS9 fed group compared to piglets in the NC and PC group. Piglets in the PC group showed significantly higher mRNA levels of proinflammatory genes IL-8 and TNF- α and anti-inflammatory gene IL-10 compared to both NC and BS9 fed group.

Next, we analysed impact of B. *subtilis*-9 supplementation on the transcript levels of genes related to intestinal cell proliferation and apoptosis. Intestinal cell proliferation associated *PCNA* gene mRNA level was significantly increased in the BS9 fed- and PC groups compared to that of the NC group (Fig. 1D). No significant difference *cyclin* gene mRNA level was observed between piglets in the NC and BS9 fed group, although significantly higher mRNA level of the *cyclin* gene was observed in the PC group.

Regarding, apoptosis related genes, no significant difference in the mRNA levels of *caspase-*3 was observed among piglets in any of the groups (Fig. 1E). Piglets in the PC group showed significantly higher mRNA levels of *Bcl-2* compared to piglets in the NC and BS9 fed group.

Next, we analysed impact of B. *subtilis*-9 supplementation on the mRNA levels of gut epithelial mucin. Results showed that, piglets in the BS9 fed group showed significantly higher mRNA levels of mucin producing gene *Muc-1* compared to piglets in both PC and NC group (Fig. 1F).

### B. *subtilis*-9 supplementation exhibits significant changes in fecal metabolic repertoires in weaning piglets

**Figure 2:**
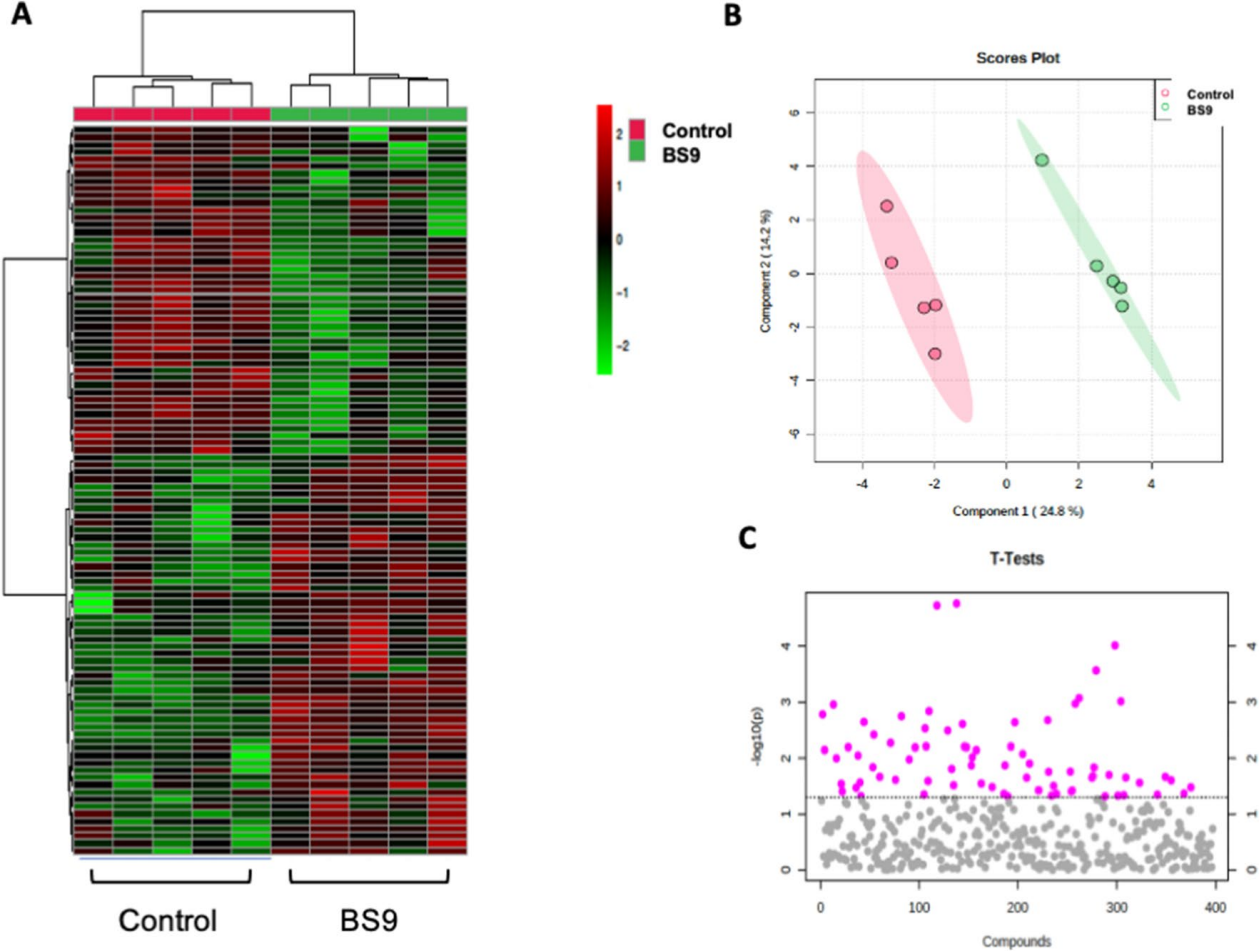
Distinct metabolic signature of the BS9 supplemented piglets. Extracellular untargeted metabolomic analyses was performed on the colon digesta samples of the piglets supplemented with BS9 vs control group, using liquid chromatography coupled to a mass spectrometer (LC–MS). (**A**) Global metabolomic profile displaying change in metabolic signature between groups, (**B**) PCA scores plot with explained variances between the selected PCs. Across the groups and (**C**) Statistical important features between the groups (n = 5) identified by t-tests, displayed in red. Statistical analysis was performed in Metaboanalyst (version 5.0) online analysis software (www.metaboanalyst.ca, Accessed 10 March 2022) using Two-sample t-tests and Wilcoxon rank-sum tests. Features with P < 0.05 were considered significant.

**Figure 3:**
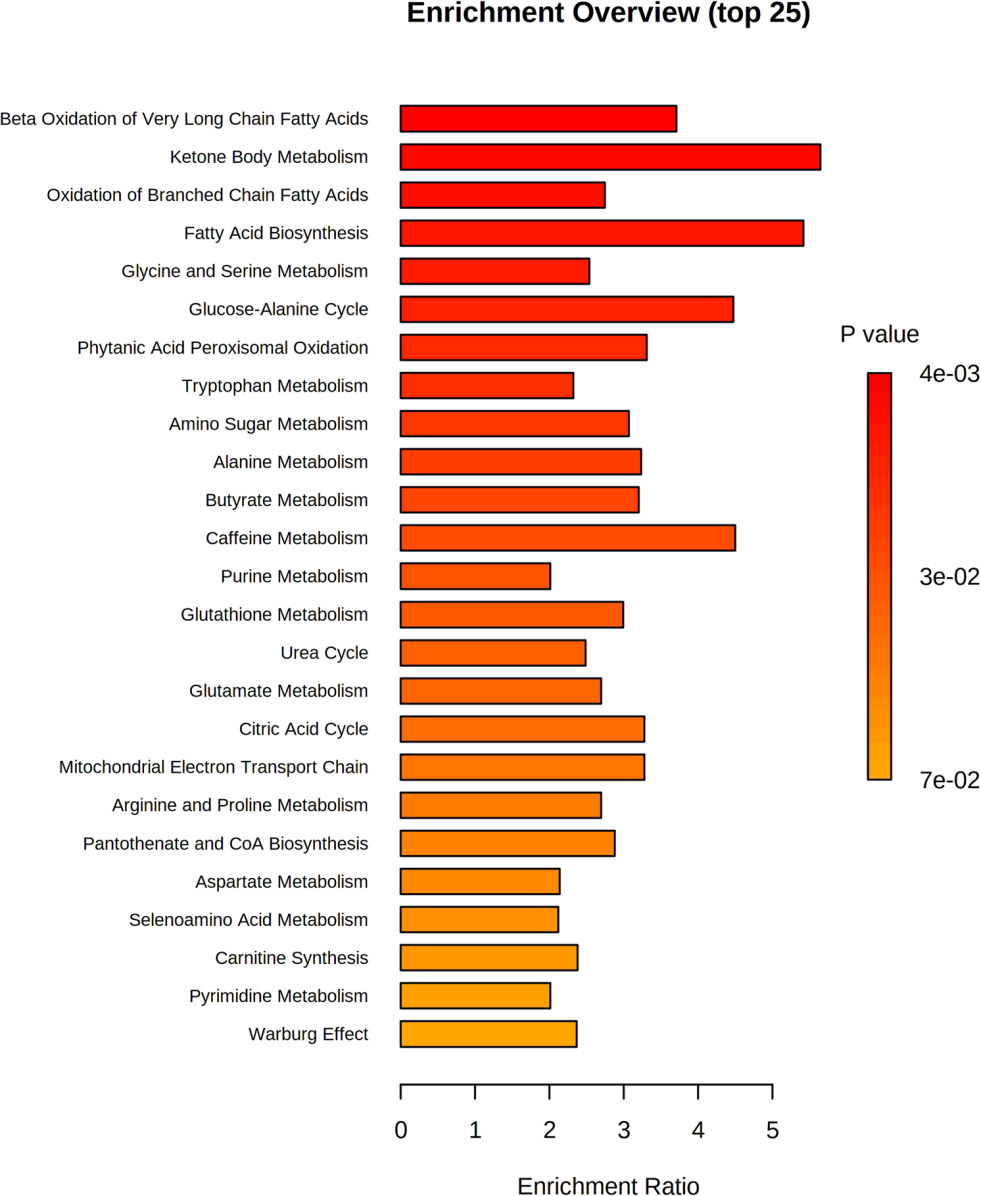
Summary plot of meaningful metabolic pathways impacted in colon digesta from the pathway sets enrichment analysis that are ranked by Holm P-value. Significant important colon digesta metabolites were analysed in Metaboanalyst (version 5.0) online analysis software (www.metaboanalyst.ca, Accessed 10 March 2022) to identify the most enriched pathways.

To decipher the metabolic impact of BS9 supplementation on weaning piglets, an untargeted metabolomic analysis was performed using LC–MS on fresh colon contents. A total of 397 metabolites were successfully identified which were then statistically analyzed through Metaboanalyst (version 5.0) online analysis software. BS9 supplementation substantially altered the metabolic profile compared to NC (Fig. 2A). To observe modulation and variation among the groups, we performed Principal Component Analysis (PCA). A distinct separation was observed between the metabolomic profiles of control diet and BS9-supplemented diet groups (Fig. 2B). Out of 74 significant differential metabolites, 40 of them increased in abundance and 34 metabolites decreased in abundance in response to BS9 supplementation (Fig. 2C and Supplement Table 3). Furthermore, we performed the quantitative pathway enrichment analysis in Metaboanalyst (Version 5) to determine the modulation in metabolic pathways upon BS9 supplementation. Results revealed that BS9 supplementation resulted in significant enrichment of 25 metabolic pathways, out of which, beta oxidation of long chain fatty acids, ketone body metabolism, oxidation of branched chain fatty acids, fatty acid biosynthesis, and glycine and serine metabolism pathways had the top five enrichment patterns (Fig. 3).

**Figure 4:**
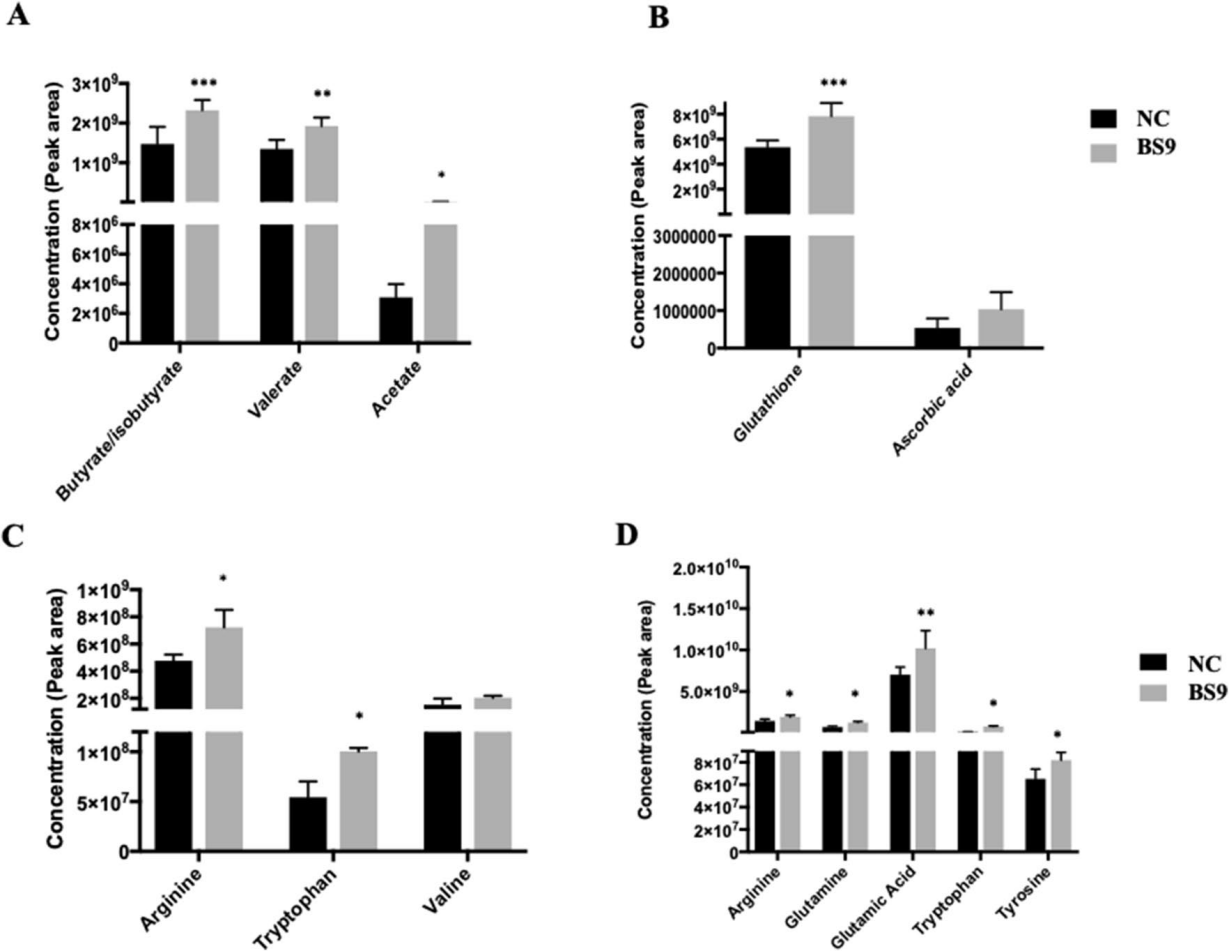
Targeted metabolomic features displaying modulation between BS9 supplemented and control group piglets. (**A**) SCFA concentration in colon digesta, (**B**) antioxidant concentration in plasma, (**C**) amino Acid concentration in colon digesta and (**D**) amino Acid concentration in plasma.

Targeted analysis revealed that BS9 supplemented group showed higher abundance of short chain fatty acids (SCFA), including butyrate, valerate and acetate in colon compared to that in the NC group (Fig. 4A). Additionally, BS9 supplementation improved the antioxidant status of the piglets as reflected by higher abundance of plasma glutathione (Fig. 4B). Finally, targeted analysis on the amino acids suggested that BS9 supplemented group had a higher abundance of arginine and tryptophan (Fig. 4C) in the colon digesta and higher abundance of arginine, glutamine, glutamic acid, tryptophan and tyrosine in plasma (Fig. 4D) compared to the control group, suggesting increased availability of certain amino acids compared to the negative control group.

## Discussion

We report the ability of the novel probiotic *B. subtilis* in improving the growth performance of the weaning piglets fed low complexity nursery diets. Our study addressed a comparative analysis to determine the efficacy of novel *bacillus*-based probiotic, BS9 as an efficient alternative to pharmacological zinc oxide. Although no differences in growth performance were reported, an increasing trend was observed compared to NC in D28, along with higher ADG of BS9-L group in Period 2. In our study, an increased ADG of the BS9 supplemented piglets is believed to be correlated with the reduction of diarrhea incidence compared to the control group. Previous studies have shown that *Bacillus subtilis* supplementation was able to reduce diarrhea and improve growth performance and interestingly, without significantly improving average daily feed intake^29,30^. This may suggest an improved feed digestibility and availability of nutrients associated with BS9 supplementation. This notion is supported by previous studies showing that *Bacillus*-based probiotic supplementation increased digestibility of crude protein and fiber in the piglets as well as growing pigs, enhancing serum amino acid levels and gross energy, which were attributed to extracellular enzyme secretion of *Bacillus* strains^11,31,32^. In our previous study, we showed that our novel *B. subtilis* strain BS9 exhibited an enhanced extracellular protease, cellulase, xylanase and amylase activities^20^. Additionally, in the same study, we also observed an enhanced digestibility of soybean with BS9 supplementation. We speculate that the increase in the body weight in BS9 supplemented piglets in this study, may be partly attributed to extracellular enzyme activity of BS9, suggesting more nutrient availability for the piglets. Our data from targeted metabolomic analysis showed a higher abundance of amino acids including tryptophan, tyrosine, glutamine and arginine in serum of the BS9 supplemented piglet, which may support this notion. However, nutrient digestibility was not analysed in this study and would need further investigation to confirm this. Additionally, contrary to the previous studies by others that observed an increase in average daily gain in early periods of supplementation^11,33,34^, we observed a delayed trend in ADG improvement reaching significant level in the later period (weeks 3–4) with no significant change in ADFI in this study. This could partly be explained via varying feeding behaviours of the piglets, feed type (such as low complexity) and feed quality such as, palatability and digestibility^35–37^.

With the increasing need on gut health in livestock industry, our study elaborated the positive impact of bacillus based probiotic supplementation on weanling piglets. Consistent with the previous studies^1,15,30^, we observed significantly lower concentrations of fecal *E. coli* and total coliforms in the BS9 supplemented piglets throughout the trial. Additionally, we also observed a higher abundance of fecal lactobacillus species in the BS9 supplemented piglets, which may explain the reduction in the fecal *E. coli* and total coliforms since, lactobacillus abundance is associated with enrichment of the gut microbiota and reduction of proteobacteria in the gut^38–40^. In light of gut health, these data are also important because higher abundance of lactobacillus in the gut is directly correlated to higher abundance of organic acids such as short chain fatty acids (SCFAs)^41–43^, as observed in our current study. Microbially driven or exogenous SCFAs such as butyrate and acetate have been shown to regulate gut homeostasis by reducing luminal pH, inhibiting pathogenic proliferation, improving nutrient digestibility in the weaning piglets and improve their growth performance^44–46^. This could further explain the growth improvement observed in our current study.

Intestinal epithelial cells turnover proliferation, differentiation, and apoptosis are essential for mucosal development, which directly influences nutrient absorption and is greatly impacted due to weaning stress, including diarrhea^47,48^. Interestingly, SCFAs such as butyrate and acetate have been shown to improve cell proliferation and reduce apoptosis in gut mucosa^49–51^. In the current study, BS9 supplementation showed elevated mRNA levels of cell proliferative gene *PCNA* compared to negative control diet group. While mRNA levels of apoptotic gene, *caspase-3* and apoptosis regulator, *Bcl-2* were not significantly affected in the BS9 supplemented group compared to the NC group, higher expression of Bcl-2 in ZnO group may suggest an escalation of programmed cell death such as apoptosis induced by pharmacological levels of ZnO. It may also suggest that BS9 and ZnO influence gut in distinct ways to induce growth improvements as observed in various studies. We acknowledge, that further tests need to be performed to quantitatively analyse the impact on cell proliferation and apoptosis, however, our gene expression and metabolomic data on SCFAs supportingly provide clues on their role(s) in explaining the observed effects of BS9 supplementation.

Intestinal integrity is critical in maintain gut health^52^, especially when morphological changes negatively impact intestinal epithelial barrier during weaning^53^. Previous studies have observed an improved expression of intestinal tight junction genes and improved epithelial barrier function upon supplementation with bacillus-based probiotics^54–56^. Higher expression of the intestinal tight junction genes in our study agrees with the previous studies and may help explaining lower diarrhea scoring in the BS9 supplemented group. However, further studies need to be performed to confirm the epithelial barrier activity as well as protein level expression of tight junction genes.

In conclusion, our study showed that supplementation with novel probiotic *B. subtilis-9* improves the body weights and gut health markers in the weaning piglets which may be attributed in parts to the reduction of diarrhea, production of SCFAs, elevation of *lactobacillus* population and augmentation of tight junction mRNA expression. Although, BS9 appeared to exhibit a different mechanism of action than ZnO, the growth performance and gut health parameters were seen comparable and, in some cases, significantly better in BS9 supplemented group. Based on the current results, the low dose supplementation can achieve significant improvements in growth performance in research setting. However, larger studies in similar setting as well as production/commercial settings need to be performed to confirm these findings. We also show *B. subtilis-9* supplementation displays a distinct metabolic signature in the weaning piglets, although its correlation with the feed efficiency was not drawn in this study. Finally, we expect that the growth outcome observed in the BS9 supplemented piglets is due to a multitude of interactions that each may have a small and synergistic effect. Our study adds knowledge to the limited body of existing research pertaining application of probiotic as an alternative to zinc oxide growth promoters and suggests that supplementation of piglets with this novel bacillus-based probiotic may improve their feed utilization efficiency and growth performance.

## Supplementary Information

Supplementary Information.

## Data Availability

The data presented in this study are available on request from the corresponding author.

## Abbreviations

AGP: Antimicrobial growth promoters
BW: Body weight
CT: Cycle threshold
CFU: Colony forming units
ADG: Average daily gain
ADFI: Average daily feed intake
FCR: Feed conversion ratio
IL-6: Interleukin 6
IL-8: Interleukin 8
IL-10: Interleukin 10
MS: Mass spectrometer
MUC-1: Mucin-1
PWD: Post-weaning diarrhea
PBS: Phosphate buffered saline
PCNA: Proliferating cell nuclear antigen
qPCR: Quantitative real time PCR
RT-PCR: Real-time polymerase chain reaction
TJP-1: Tight junction protein-1
TLR-2: Toll like receptor 2
TLR-4: Toll like receptor 4
TNF-α: Tumor necrosis factor- α
UHPLC: Ultra high-performance liquid chromatography
ZnO: Zinc oxide
