# Supplementary material for: Comparative efficacy of a novel Bacillus subtilis-based probiotic and pharmacological zinc oxide on growth performance and gut responses in nursery pigs
Source: Sci Rep. 2023 Mar 22;13:4659. doi: 10.1038/s41598-023-31913-0 (PMC10033705; doi:10.1038/s41598-023-31913-0)
Supplement: Supplementary file 1 — Supplementary Information. [file 41598_2023_31913_MOESM1_ESM.pdf]

Comparative Efficacy of a Novel *Bacillus subtilis*-based Probiotic and Pharmacological Zinc Oxide on Growth Performance and Gut responses in nursery Pigs

Sudhanshu Sudan[1], Lauren Fletcher[1], Xiaoshu Zhan[1], Serena Dingle[1], Rob Patterson[3], Lee-Anne Huber[1], Robert Friendship[2], Elijah G. Kiarie[1], and Julang Li[1].

[1]Department of Animal Biosciences, University of Guelph, Ontario, Canada

[2]Department of Population Medicine, University of Guelph, Ontario, Canada

[3]CBS Bio Platforms Inc., Calgary, Alberta, Canada

Table S1 Composition of experimental diets (as-fed basis)

Item	Phase 1		Phase 2	
	PC	NC	PC	NC
Corn	46.28	46.67	56.46	56.85
Soybean meal 46%	25.39	25.39	27.59	27.56
Wheat	10.00	10.00	10.00	10.00
Whey permeate	8.00	8.00		
Fishmeal	5.00	5.00		
Soy oil	2.00	2.00	2.00	2.00
L-Lysine HCL	0.42	0.42	0.43	0.43
DL-Methionine	0.15	0.15	0.14	0.14
L-Threonine -98%	0.07	0.07	0.13	0.13
L-Tryptophan-98%	0.02	0.02	0.01	0.01
Limestone	0.81	0.81	1.01	1.01
Monocalcium phosphate	0.67	0.67	0.97	0.97
Vitamin and trace mineral premix[1]	0.60	0.60	0.60	0.60
Salt	0.20	0.20	0.30	0.30
Zinc oxide 72%	0.39		0.39	
Calculated provisions				
Net energy, Kcal/kg	2505	2515	2494	2505
Crude protein, %	20.8	20.8	19.0	19.0
Crude fat, %	4.43	4.44	4.36	4.38
Crude fibre, %	2.51	2.51	2.77	2.78
Sid Lys, %	1.35	1.35	1.23	1.23
SID Met, %	0.47	0.47	0.41	0.41
SID Met + Cys, %	0.74	0.74	0.68	0.68

SID Try, %	0.22	0.22	0.20	0.20
SID Thr, %	0.73	0.73	0.73	0.73
SID Val, %	0.83	0.83	0.77	0.77
Std. Dig. Phosphorous, %	0.40	0.40	0.33	0.33
Calcium, %	0.80	0.80	0.70	0.70
Total Phosphorous, %	0.59	0.59	0.53	0.53
Sodium, %	0.17	0.17	0.14	0.14
Chloride, %	0.26	0.26	0.29	0.29
Zinc, mg/kg	3000.00	141.04	3000.00	144.34
Copper, mg/kg	19.97	19.98	20.35	20.36

Dietary treatments: PC, positive Control (with 0.39% Zinc Oxide72); NC, Negative Control (without Zinc Oxide); Phase I (0–7 d post-weaning) and phase II (d8–28 d post-weaning).

[1] Provided per kg of premix: vitamin A, 2,000,000 IU as retinyl acetate; vitamin D3, 200,000 IU as cholecalciferol; vitamin E, 8,000 IU as dl-α-tocopherol acetate; vitamin K, 500 mg as menadione; pantothenic acid, 3,000 mg; riboflavin, 1,000 mg; choline, 100,000 mg; folic acid, 400 mg; niacin, 5,000 mg; thiamine, 300 mg; pyridoxine, 300 mg; vitamin B12, 5,000 mcg; biotin, 40,000 mcg; Cu, 3,000 mg from CuSO4×5H2O; Fe, 20,000 mg from FeSO4; Mn, 4,000 mg from MnSO4; Zn, 21,000 mg from ZnO; Se, 60 mg from Na2SeO3; and I, 100 mg from KI (DSM Nutritional Products Canada Inc., Ayr, ON, Canada).

Table S2. Genes and primer sequences used for quantitative real time PCR.

Gene	Primer sequence
TLR2	F-ACGTATCCATCAATGAACACTGC<br>R-GTCCGTTAAGGGTGCAGTCA
TLR4	F-GCCATCGCTGCTAACATCATC<br>R-CTCATACTCAAAGATACACCATCGG
TLR9	F-CACGACAGCCGAATAGCAC<br>R-GGGAACAGGGAGCAGAGC
TNFα	F-GGCCCAAGGACTCAGATCAT<br>R-GGCATACCCACTCTGCCATT
IL-6	F-CCCTGAGGCAAAAGGGAAAGAA<br>R-CTCAGGTGCCCCAGCTACAT
IL-8	F-TTCGATGCCAGTGCATAAATA<br>R-CTGTACAACCTTCTGCACCCA
IL-10	F-TGCATCCACTTCCCAACCAG<br>R-GGCAACCCAGGTAACCCTTA
MUC1	F-CTCTGCTCAGCCTGGGTCT<br>R-GCTACATAGGATGGTAGGCA
Claudin-1	F-AGCTATGGCCAACGCGG<br>R-TGCTTGCAAAGTGGTGTTCAG
Occludin	F-ATCAACAAAGGCAACTCT<br>R-GCAGCAGCCATGTACTCT
Zona occludens-1	F-GAGTTTGATAGTGGCGTT<br>R-GTGGGAGGATGCTGTTGT
GAPDH	F-AGCAATGCCTCCTGTACCAC<br>R-AAGCAGGGATGATGTTCTGG

Table S3 Significant change in the metabolites emerging in BS9 supplemented weaning piglets comparted to negative control group as assessed by Welch's two-sample *t*-test.

Metabolite	t.stat	p.value
**Diaminopimelic acid**	-9.0976	1.7118e-05
**Arginine**	-8.9987	1.8551e-05
**2-[4-(3-Amino-2-hydroxypropoxy)phenyl]acetamide**	-7.1564	9.6485e-05
**indole-3-acetic acid**	-6.1605	0.00027087
**2,4-Dihydroxybenzoic acid**	-5.1764	0.00084645
**3-(1-hydroxyethyl)-2,3,6,7,8,8a-hexahydropyrrolo[1,2-a]pyrazine-1,4-dione.1**	-5.0696	0.00096547
**3-Methylsalicylic acid**	-4.9912	0.0010645
**Valylproline**	-4.9643	0.001101
**3-Phenyllactic acid**	4.7487	0.0014476
**Alanine**	4.6477	0.0016496
**D-_-Hydroxyglutaric acid**	4.5891	0.0017806
**Hypoxanthine.1**	-4.4647	0.002098
**N-Acetylalanine**	4.4081	0.0022623
**Catechol.1**	-4.3994	0.0022885
**2-Hydroxy-4-(4-hydroxyphenyl)butanoic acid**	4.3465	0.0024568
**1,5-Anhydro-D-glucitol**	4.2137	0.0029409
**L-Alanyl-L-proline**	-4.1482	0.0032169
**2-Hydroxycaproic acid**	4.0258	0.00381
**4-Methyl-5-thiazoleethanol**	3.7852	0.0053485
**Methionine sulfoxide**	3.6876	0.0061512
**Valerate**	-3.6819	0.0062025
**Capryloylglycine**	3.6788	0.0062296
**Succinic acid**	3.6638	0.0063664
**Acetyl-L-carnitine**	3.6608	0.0063939
**Imidazolelactic acid**	3.6558	0.0064399
**Alanine.2**	3.5829	0.0071577
**Dodecanedioic acid**	3.5764	0.0072256
**Hexanoic acid**	-3.4745	0.0083874
**4-Oxoproline**	3.4286	0.0089733

**N-Acetyl-_-D-glucosamine**	3.3846	0.0095756
**Catechol**	-3.3533	0.010031
**Xanthine**	3.3208	0.010528
**4-Ethylphenol**	-3.2076	0.012468
**MAB-CHMINACA metabolite M3**	3.1589	0.013418
**Pantothenic acid**	3.158	0.013436
**Asparagine**	3.1095	0.014456
**Pyridoxamine.1**	-3.1027	0.014606
**N6-Acetyl-L-lysine**	3.0614	0.015549
**Hydrocinnamic acid.1**	-2.9889	0.017363
**Hypoxanthine.2**	-2.9836	0.017505
**Azelaic acid.2**	-2.9	0.019891
**3-(4-Hydroxyphenyl)propionic acid.1**	-2.8694	0.020848
**N-Acetylneuraminic acid**	2.8488	0.021517
**2'-Deoxyinosine**	-2.848	0.021546
**_-Asarone**	-2.8302	0.022144
**Phenylacetaldehyde**	-2.8265	0.022269
**7-Methylxanthine**	-2.8247	0.022332
**4-Acetamidobutanoic acid**	2.7688	0.024342
**Adenosine.1**	-2.7555	0.024848
**3-(4-Hydroxyphenyl)propionic acid**	-2.7373	0.025556
**4-Oxoproline.1**	2.6977	0.02717
**Amobarbital**	-2.6908	0.027461
**_-L-Glutamyl-L-glutamic acid**	2.6697	0.028375
**Uracil**	2.6606	0.028777
**N6,N6,N6-Trimethyl-L-lysine**	2.6239	0.030467
**4-Methyl-5-thiazoleethanol.1**	-2.6066	0.031293
**N-Acetylneuraminic acid.1**	2.5762	0.032812
**1-(1,3-Benzodioxol-5-yl)-N-{[(2R,4S,5R)-5-vinyl-1-azabicyclo[2.2.2]oct-2-yl]methyl}methanamine**	-2.5699	0.03313
**1-Methyl-3,6-(1H,2H)-pyridazinedione**	2.5583	0.033734
**Heptanoic acid**	-2.4906	0.037487
**Guanine.2**	-2.4847	0.037832
**Guanine.1**	-2.4626	0.03916
**Proline**	2.458	0.039443

**Hexadecanedioic acid**	-2.4038	0.04292
**Nalbuphine**	2.3979	0.04332
**1-Aminocyclohexanecarboxylic acid.1**	2.3946	0.043539
**DL-Carnitine.1**	2.3797	0.044567
**Thymidine**	-2.3746	0.044922
**Tryptophan.1**	-2.361	0.045888
**2,4-Diaminoanisole**	-2.3514	0.046578
**Nikethamide 1-oxide**	-2.3486	0.046783
**Pipecolic acid**	2.3374	0.047608
**Indole-3-acrylic acid**	-2.3324	0.047983
**_-Valerolactam.1**	-2.3247	0.048562
